# Supplementary material for: Gene Gangs of the Chloroviruses: Conserved Clusters of Collinear Monocistronic Genes
Source: Viruses. 2018 Oct 20;10(10):576. doi: 10.3390/v10100576 (PMC6213493; doi:10.3390/v10100576)
Supplement: Supplementary file 1 [file viruses-10-00576-s001.zip › viruses-363410-suppl_/supplementary/File_S7.zip!/plots_subtracted_combo/Bin_conservation_minus_background_CVR-1_vs_MA-1E.pdf]

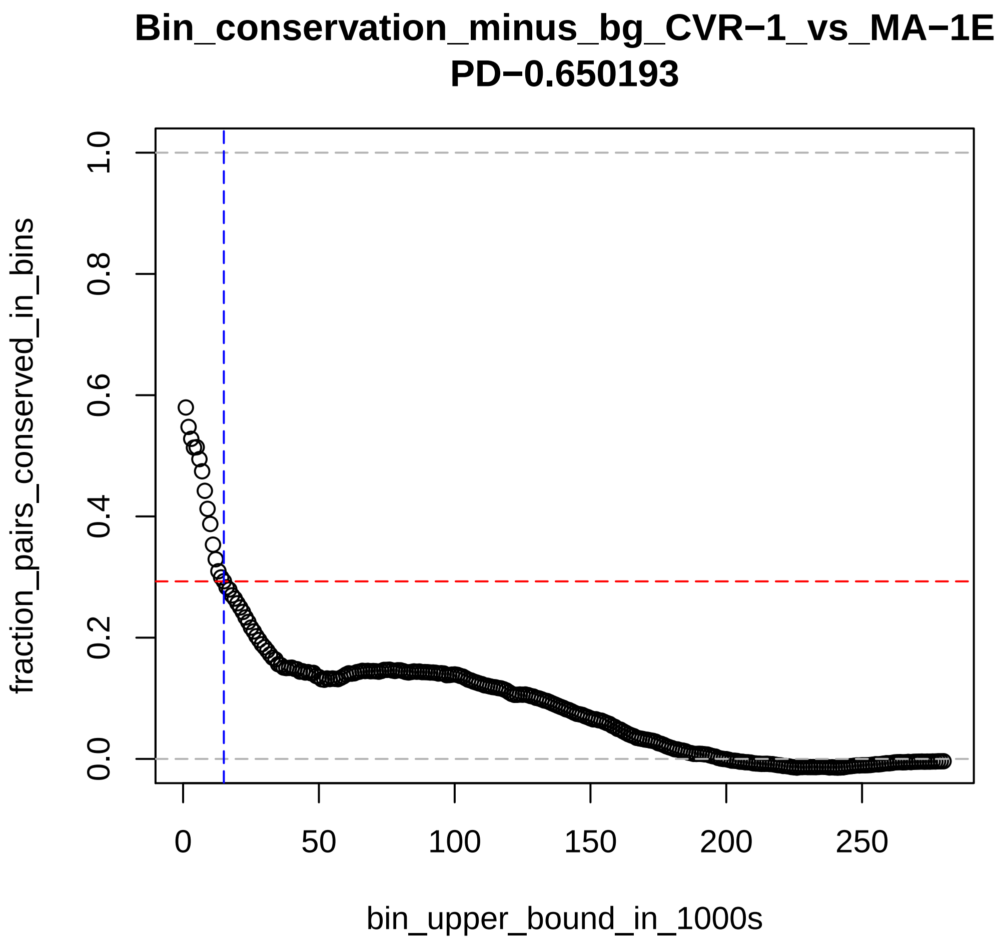

Bin_conservation_minus_bg_CVR−1_vs_MA−1E
PD−0.650193
fraction_pairs_conserved_in_bins
0.0
0.2
0.4
0.6
0.8
1.0
0
50
100
150
200
250
bin_upper_bound_in_1000s

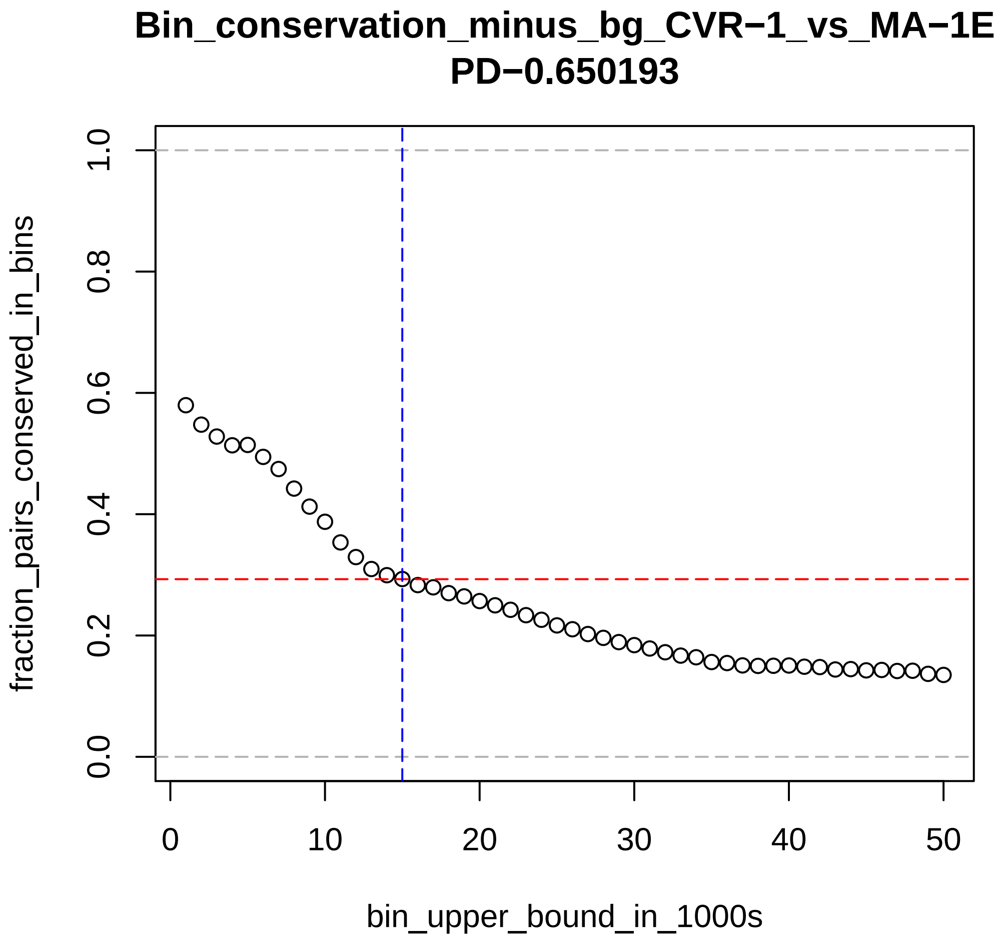

Bin_conservation_minus_bg_CVR−1_vs_MA−1E
PD−0.650193
fraction_pairs_conserved_in_bins
0.0
0.2
0.4
0.6
0.8
1.0
0
10
20
30
40
50
bin_upper_bound_in_1000s
